# P^2^ESA: Privacy-Preserving Environmental Sensor-Based Authentication

**DOI:** 10.3390/s25154842

**Published:** 2025-08-06

**Authors:** Andraž Krašovec, Gianmarco Baldini, Veljko Pejović

**Affiliations:** 1 Joint Research Centre, European Commission, Via Enrico Fermi 2749, 21027 Ispra, Italy; gianmarco.baldini@ec.europa.eu; 2 Faculty of Computer and Information Science, University of Ljubljana, Večna pot 113, 1000 Ljubljana, Slovenia; veljko.pejovic@fri.uni-lj.si

**Keywords:** behavioural authentication, edge computing, privacy-preserving learning, ubiquitous sensing

## Abstract

The presence of Internet of Things (IoT) devices in modern working and living environments is growing rapidly. The data collected in such environments enable us to model users’ behaviour and consequently identify and authenticate them. However, these data may contain information about the user’s current activity, emotional state, or other aspects that are not relevant for authentication. In this work, we employ adversarial deep learning techniques to remove privacy-revealing information from the data while keeping the authentication performance levels almost intact. Furthermore, we develop and apply various techniques to offload the computationally weak edge devices that are part of the machine learning pipeline at training and inference time. Our experiments, conducted on two multimodal IoT datasets, show that P^2^ESA can be efficiently deployed and trained, and with user identification rates of between 75.85% and 93.31% (c.f. 6.67% baseline), can represent a promising support solution for authentication, while simultaneously fully obfuscating sensitive information.

## 1. Introduction

Alice is employed as a caregiver in an assisted living facility. While working at a shared computer terminal used for managing residents’ medical data, Alice is contacted by her colleague Bob, who asks her to join him for a cup of coffee in the lobby. To avoid the hassle of re-authentication, Alice leaves for a quick break without logging off. Seeing that Alice is gone, Carl sneaks into the room and takes over the session at the terminal. Such access enables Carl complete access to patients’ sensitive data to be misused, altered, or encrypted for Carl’s personal gain.

Digital technologies revolutionised access control to both physical and virtual resources. From authentication based on something that a user has (e.g., an RFID key fob), we advanced towards using something that a user knows (e.g., a password), and even harnessing something that is an inherent property of a user (e.g., a person’s biometrics). However, as we entrust these methods with mediating access in highly dynamic scenarios, we observe that the current means of authentication are neither unobtrusive nor continuous, and are thus vulnerable to “coffee-break attacks” such as the one described above.

The drawbacks of existing methods are apparent. For example, key fobs can be lost or stolen. Biometrics, while suitable for one-time authentication, are cumbersome for frequent identity verification. Finally, misuse of passwords is already well documented and is best illustrated by a recent study of authentication practices in hospitals, which found that codes for access to emergency care rooms are posted on room doors because preventing authentication due to a forgotten code can have fatal consequences [1]. While most modern authentication systems rely on multiple authentication factors, the inherent problems of individual approaches remain.

As an alternative, behavioural biometrics harness the relative uniqueness of human behaviour and match the observed behaviour with pre-collected patterns of known users. The proliferation of the Internet of Things (IoT) devices with sensing capabilities—indeed, it is estimated that approximately 30 billion such devices will be in use by 2030 [2]—means that the data reflecting a user’s behaviour is widely available. Recently, authentication based on sensor data from sources ranging from PC usage patterns, over computer-mouse movements, to the way drawers are opened in a room, have been demonstrated [3,4,5]. While behaviour-based authentication does not yet provide the robustness of properly used traditional authentication methods, it has been shown to achieve very high reliability in continuous authentication scenarios [6].

However, the data required for such a system to operate may present a significant privacy risk. While hashed passwords can be used for a single purpose only—user authentication—multimodal sensor data can be used for numerous malicious purposes. For instance, a third party can use the data not only to authenticate a user but also to learn about a user’s behavioural preferences, thus building a profile of a user’s activities, which can then be used for targeted advertising. Furthermore, sending a “hash” of IoT-sensed data is unlikely to lead to reliable authentication as, unlike passwords, behaviour patterns of a user need not be exactly the same every time authentication is attempted. Consequently, the identification on the server has to go beyond simple matching and harness more sophisticated machine learning classifiers instead. In summary, to enable large-scale practical use of behavioural biometric-based authentication solutions, we need to ensure that any data leaving a user’s trusted local environment carries information relevant for authenticating a user, yet excludes information that is deemed sensitive. In this research, we hypothesise that it is indeed possible to develop a practical system that reveals the information required for user authentication while hiding the information that the user wishes to protect, without significantly compromising authentication accuracy.

In this paper, we present P^2^ESA, a **p**rivacy-**p**reserving **e**nvironmental **s**ensor-based **a**uthentication method that harnesses behavioural biometrics. The core of P^2^ESA is a “filter” that passes identification-relevant information collected by IoT sensors to the authenticating authority, yet keeps selected sensitive information from leaving the trusted local environment. For this, P^2^ESA relies on adversarial learning and a split implementation of an encoder–classifier architecture, where the encoder part resides fully in the local environment. Unlike the existing approaches (e.g., [7]), P^2^ESA employs a novel iterative training that results in a robust encoder that prevents an adversary from retraining their malicious classifiers to circumvent the obfuscation brought by the P^2^ESA encoder. Furthermore, unlike its predecessors [7,8], P^2^ESA enables split operation even at training time instead of distributing the encoder to the edge devices after the training is completed. In return, no raw data leaves the local environment at any point of the process. The discrepancy between the computational capabilities of the authenticator (e.g., a third-party server) and a device conducting local data collection (e.g., a single-board computer) can render the split learning prohibitively slow. To overcome this major challenge, in P^2^ESA we also propose a set of engineering improvements to make the distributed training practical. Finally, to demonstrate the usability of behaviour-based authentication with P^2^ESA, we perform experiments with various IoT-based datasets, including multimodal sensor data collected from 15 users in an office-like environment and data collected from another group of 15 users equipped with wearable sensing devices. Our results show that P^2^ESA can be efficiently deployed and trained, and with user identification rates of between 75.85% and 93.31% (c.f. 6.67% baseline), represents a promising support solution for authentication, while simultaneously fully obfuscating sensitive information.

## 2. Related Work

Behavioural biometric authentication emerged as an alternative to traditional biometric-based authentication that relies on potentially sensitive fingerprint, iris scan, and facial image data [9]. Instead, behavioural biometrics harness the fact that a user’s behaviour often gets reflected in what is considered non-sensitive data collected by unintrusive ubiquitous sensors. To date, behavioural biometric solutions relying on various sources of data, including interaction patterns collected off touchscreens, keyboards, and mice [10,11], gait dynamics captured by wearable accelerometers and gyroscopes [12], and even soft sensors, such as PC and smartphone usage reports [3,13], have been demonstrated.

The uptake in behaviour biometric-based solutions is partly driven by users’ perception of IoT-sensed data being less privacy-sensitive than the traditional biometric data [14]. Yet, these seemingly benign data can be misused for unintended purposes. Thus, the keystroke patterns from an on-keyboard accelerometer can be used for both user identification, but also to reveal what a person is typing [15]. Similarly, data gathered for gait-based authentication can also be used to detect the activities a user is performing.

To combat these information leakages, adversarial learning, in combination with deep learning (DL), is often used. Adversarial learning harnesses separate DL modules that compete to achieve opposing goals, such as generating realistic yet fake data and discriminating between fake and real data [16]. In the context of privacy-preserving behavioural biometrics, the opposing goals are (1) user identification and (2) inference of other identifiable factors (e.g., person’s current task, emotional state, etc.), where the adversarial learning should prevent (2) while concurrently enabling (1).

Recently, adversarial learning-based approaches specifically tailored for sensor data obfuscation have appeared. Malkezadeh et al. [17] propose an encoder trained with a multi-objective loss function that minimises the ability of an attacker to unpick the undesired information, yet at the same time suppresses signal distortion, thus preserving the utility of the signal. While the use of the encoder remains local, the proposed encoder training requires that the data is fully available on the cloud. In our work, we keep all the data local, and no raw data ever reaches the cloud. The use of local obfuscation was proposed in a workshop paper by [18]. However, only the conceptual framework was presented, and, unlike this work, no real-world implementation and evaluation were provided. Privacy adversarial network (PAN) introduces an adversarial classifier and a signal reconstructor that aim to infer the hidden information and reconstruct the signal from the encoding [7]. We base our solution on the modularity and the multistep training procedure presented in PAN. However, in this work, we discard the reconstructor, thus avoiding sending raw data to the server even in the training phase. DeepObfuscator presents a solution similar to PAN, yet specifically targets image obfuscation by leveraging multiscale structural similarity in the adversarial loss function [19]. This, however, restricts the generalisability of the solution, an aspect that is addressed by Olympus [20]. Olympus enables multi-target obfuscation of information on different types of gathered data while limiting the negative effect on the functionality and generalisability of their application. Blinder [21] is another anonymisation approach that relies on variational autoencoders and obfuscates data even in a case of uneven distribution of the training dataset. We summarise different approaches in Table 1.

Finally, as noted by Hajihassani et al. [22], the attribute inference attack, where the attacker passes sensor data with known labels through the encoder, (re-)trains the model on the obfuscated data, and uses this model to reveal private attributes from a new batch of user’s data, has not been analysed in either of the mentioned state-of-the-art approaches. In this paper (Section 5), we show that such an attack completely reverses the obfuscation provided by PAN and develop a novel iterative training procedure that successfully prevents the attribute inference attack.

There are alternative approaches to preserve privacy in behaviour-based user authentication systems. Federated learning (FL) is the most prominent alternative that decentralises the machine learning approach by collaboratively training a shared model while the data of individual clients remains in their local environment. Coelho et al. [23] present an FL-based authentication system based on multimodal biometric data from medical records. Cai et al. [24] even rely on merging FL with contrastive learning, which trains models to differentiate between similar and dissimilar data samples. While these and similar works [25,26] present favourable results with a high retention of privacy, there are significant downsides, such as the large communication overhead and potentially slow model convergence, especially in the case of a large number of clients and heterogeneous non-independent and identically distributed datasets. Furthermore, these solutions do not cater to our scenario where the multimodal sensor data are aggregated at a single local device, not a federation of devices, nor do these solutions ensure protection against the the attribute inference attack mentioned above.

## 3. Threat Model

**Figure 1:**
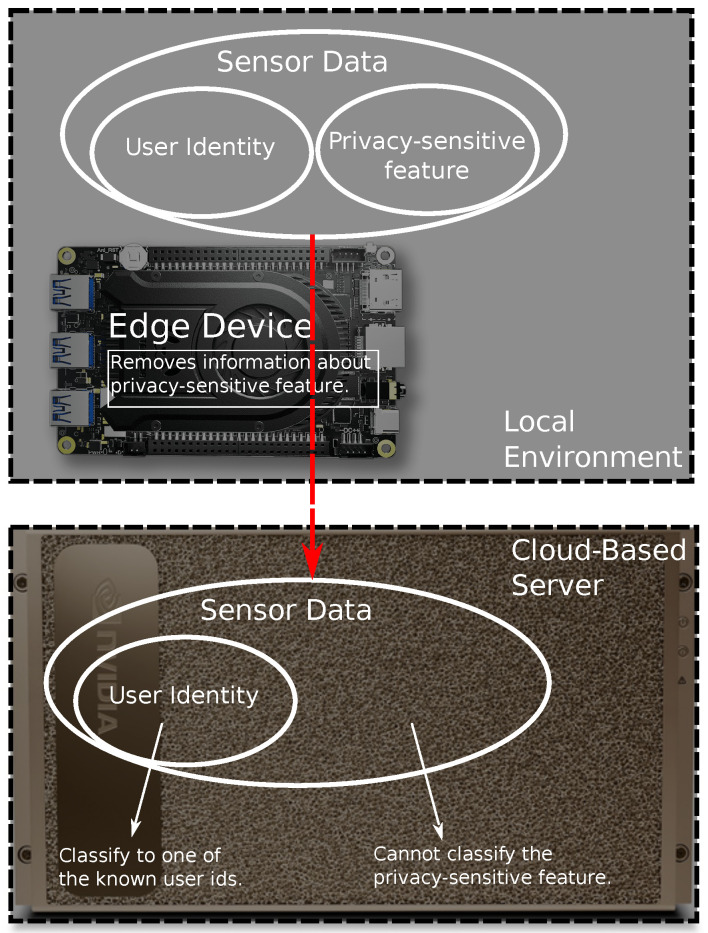
An overview of the designed functionality of P^2^ESA. The sensed data includes information about both, user’s identity, as well as the information we would like to keep private. This information is removed before the data leaves the local environment, and it cannot be inferred on the cloud (server) side.

We assume that the cloud-based server is an honest-but-curious participant whom we want to prevent from revealing potentially sensitive information from the user-controlled environment. The nature of the sensitive information (e.g., whether it is a user’s activity, emotion, etc.) is known in advance to all parties involved. We further assume that the cloud-based server is capable of architecting and training deep learning models for inferring sensitive information. We presume that the server is honest, in the sense that the distributed DL training is conducted according to the algorithm detailed in Section 4. An outline of the architecture is displayed in Figure 1.

As stated in the previous section, we also consider the possibility of an attribute inference attack. Thus, we assume that the adversary can “push” data with known labels through the encoder and can re-train the server-side classifiers with the new information. In practice, this can occur when, for example, privacy leakage happens through a side channel revealing the labels of the past data, or when the encoder structure and weights are revealed. Finally, to ensure the identification part of the proposed solution works, we assume that the server-side classifier knows the existence of all the user IDs upfront and that all of them are observed in the training set. Such a threat model is aligned with models presented in the related work [20,21].

Formally, we define privacy as the probability of the attacker correctly inferring the class that the sensitive information we try to conceal belongs to [7]. Let *X* be a dataset of sensor records, while $Y_{u}$ and $Y_{t}$ represent the user identity labels and the labels of the sensitive property we aim to protect, respectively. We define the attacker’s prediction as the sensor data first processed by the encoder (*E*) and then by the classifier ($C_{t}$) aiming to uncover the protected data: $C_{t}\left(E\left(X\right)\right)\to Y_{t}^{\prime }$. We denote the inference of the private attribute of a single data instance as $y_{t}^{\prime }\in Y_{t}^{\prime }$. To consider a system successful with respect to the concealment of the private attribute, the rate of correctly inferred labels should remain roughly equal to the predictions made by the naive classifier, which is $1/N_{t}$, where $N_{t}$ represents the number of distinct classes of the sensitive-feature attribute:

(1) $$\frac{1}{|Y_{t}|}\sum_{i=0}^{|Y_{t}|}1_{\left\{y_{t_{i}}=y_{t_{i}}^{\prime }\right\}}\approx \frac{1}{N_{t}}$$

Simultaneously to selectively blocking the information flow and ensuring that the privacy is preserved, P^2^ESA also aims to let enough information through, so that a user identification classifier ($C_{u}$) that relies on IoT sensor data can authenticate a user substantially better than a random guess, i.e.:

(2) $$\frac{1}{|Y_{u}|}\sum_{i=0}^{|Y_{u}|}1_{\left\{y_{u_{i}}=y_{u_{i}}^{\prime }\right\}}>>\frac{1}{N_{u}}$$

## 4. P^2^ESA

**Figure 2:**
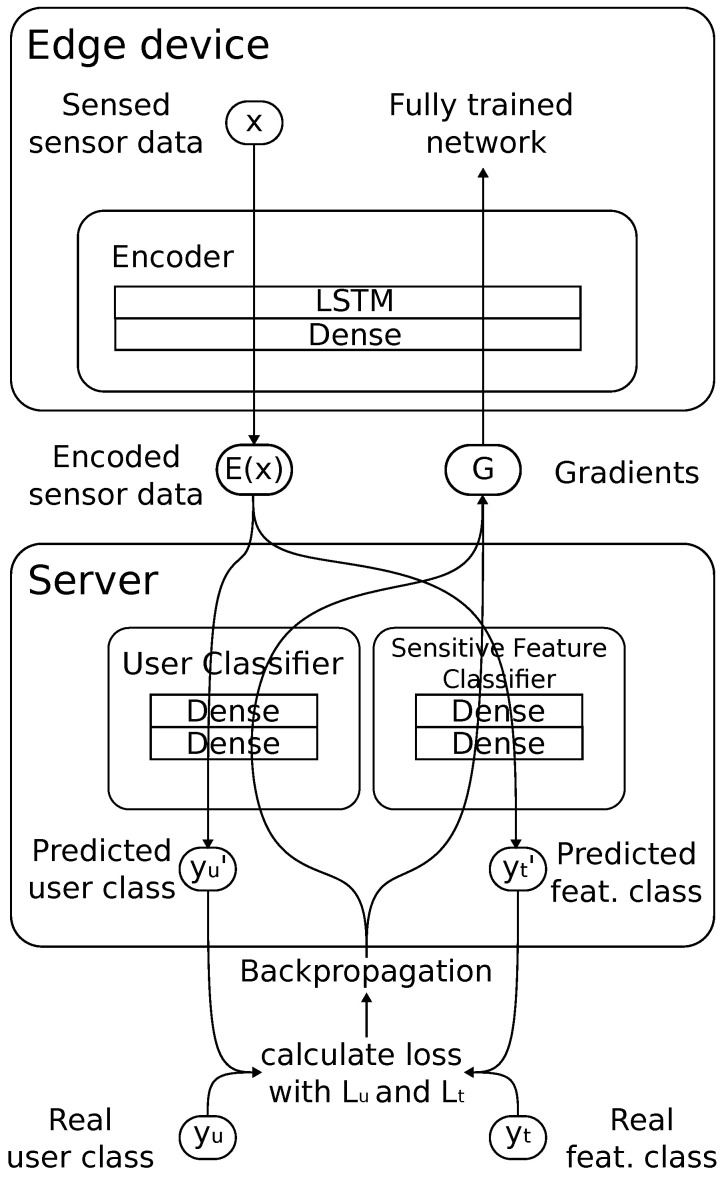
P^2^ESA architecture. The network is split over the edge device and the server, where the encoder resides on the edge device, while the classifiers reside on the (cloud) server. P^2^ESA ensures that the encoded sensor data that leaves the local environment allows for user identification through the user classifier, yet prevents sensitive information inference through the sensitive-feature classifier.

In this section, we present P^2^ESA, a framework for privacy-preserving authentication based on IoT sensor data. At the core of P^2^ESA are the underlying split neural network architecture (Figure 2) and a novel adversarial training algorithm. The combination of these concepts enables P^2^ESA to achieve selective data obfuscation in an efficient, distributed manner. The architecture consists of three modules: (i) the encoder, (ii) the user classifier, and (iii) the sensitive-feature classifier, residing across two entities—an edge device and a server. In the remainder of this section, we detail the three modules and the distributed training procedure.

### 4.1. Data-Obfuscating Encoder

The purpose of the encoder is to act as a filter that prevents raw sensor data from leaving the local environment and ensures that the data sent to a server avails user identification, yet hides a predefined aspect of user behaviour, be it a task a user is currently performing, a user’s emotional state, demographics, or other factors. The encoder resides in the users’ local environment and any sensed data has to pass through the encoder, where it is obfuscated, before it is forwarded to the server. In essence, the encoder acts like a dimensionality reducer, as it takes multimodal data and converts them to a lower-dimensional embedding. In practice, the non-linearity of the encoder network together with the adversarial training procedure employed by P^2^ESA results in an embedding that is free from information that reveals a selected hidden property (e.g., a task a user is performing, emotional state, etc.), yet preserves enough information to allow user identification. Theoretical guarantees of such obfuscation, on the other hand, are further discussed in Section 6.

Edge devices are often limited in their computational capabilities, available memory, and power consumption. Thus, the main practical challenge is to develop a lightweight encoder so that it can be trained on an edge device while having enough capacity to successfully transform the gathered sensor data into an embedding that allows for user identification, yet obfuscates sensitive information. Based on extensive empirical experimentation with different edge devices (Raspberry PI 3B, Raspberry Pi Ltd, Cambridge, UK, LattePanda 3 Delta, LattePanda, Shenzen, China) and different datasets (described in Section 5), in this work, we model the encoder as a neural network consisting of a single LSTM layer with 128 nodes, followed by a single fully-connected layer with 128 nodes. We note, however, that other architectures may prove more suitable for future, potentially more powerful, edge devices. Furthermore, in this work, we deploy the encoder on a single edge device, yet we foresee a potential horizontal distribution of the encoder across multiple devices in the future.

### 4.2. User Classifier

The user classifier resides on a server outside local control. This classifier is a part of the “legitimate” flow of information and is tasked with authentication, i.e., differentiating among different users based on the encoded data sent from the local IoT environment. In this paper, we aimed to keep this classifier lightweight as well; thus, in our implementation, it consists of three fully connected layers with 64, 32, and $N_{u}$ nodes, respectively, where $N_{u}$ represents the number of users in the dataset. In between the fully connected layers, the dropout layers with a dropout ratio of 0.3 are added to prevent overfitting. We utilise the rectified linear unit (ReLU) activation function for all layers, bar the last one, where we use softmax to obtain predicted probabilities of user classes. The user class with the highest probability is predicted as the user currently present in the environment.

### 4.3. Sensitive-Feature Classifier

The sensitive-feature classifier also resides on a (remote) server. This classifier, however, takes the role of an adversary and aims to infer user or environment characteristics that should remain hidden. In our experiments (Section 5), such characteristics include a user’s current task or their emotional state. In our implementation, we use an identical architecture for both the user and the sensitive-feature classifier, except for the number of nodes on the final layer ($N_{t}$), as this number depends on the number of different labels the classifiers is designed to distinguish among.

### 4.4. Multistep Adversarial Training Process

To train our split network architecture, we develop an adversarial training algorithm (Algorithm 1) that not only trains all components of the system but also ensures that no raw data leaves the local network, even at training time. The goal of the process is to train the encoder in a way that information about a user’s identity is sent through, while other (predetermined) aspects, e.g., current task or user mood, are filtered out. In each epoch of the training, we perform the following three steps:

1. Legitimate data flow—training of the encoder and user classifier—detailed in Section 4.4.1.
2. Adversarial data flow—sending the data through the fixed encoder and training the sensitive-feature classifier—detailed in Section 4.4.2.
3. Encoder feedback—training the encoder by weighing and combining the value of loss functions of both server modules—detailed in Section 4.4.3.

**Algorithm 1** The P^2^ESA training algorithm.  1:Initialise $\theta_{e}$, $\theta_{u}$, $\theta_{t}$  2:**repeat**  3:      **for** epoch in 1,2 … Epochs **do**  4:            **for** minibatch in X **do**  5:                   Forward pass to calculate $L_{u}$  6:                   $\theta_{u}\leftarrow$ backpropagation with $L_{u}$  7:                   $\theta_{e}\leftarrow$ backpropagation with $L_{u}$  8:                   Forward pass to calculate $L_{t}$  9:                   $\theta_{t}\leftarrow$ backpropagation with $L_{t}$10:            **end for**11:            **for** minibatch in D **do**12:                  Forward pass to calculate $L_{u}$ and $L_{t}$13:                  $\theta_{u}\leftarrow$ backpropagation with $L_{u}$14:                  $\theta_{e}\leftarrow$ backpropagation with $L_{e}$15:            **end for**16:      **end for**17:      Initialise *a*18:      **for** epoch in 1,2 … (Epochs×3) **do**19:            **for** minibatch in D **do**20:                  Forward pass to calculate $L_{t}$21:                  $\theta_{t}\leftarrow$ backpropagation with $L_{t}$22:            **end for**23:            acc← epoch classification accuracy24:            a←[a,acc]25:      **end for**26:**until** $acc<\bar{a}+\epsilon$▹ models’ parameters

We utilise all mini-batches of data within an epoch to perform the first two steps and only then apply the same mini-batches to perform the last step. This helps the system to converge faster as both classifiers are better trained before providing the feedback to the encoder in the last step.

Once the three-step process is complete, we further refine the task classifier, which is detailed in Section 4.4.4. The training is halted when additional task classifier training yields no significant improvement in terms of sensitive-feature classification accuracy.

#### 4.4.1. Legitimate Data Flow

The first training step in every epoch simultaneously trains the user classifier and the encoder, essentially teaching the encoder to relay necessary information to the server. Therefore, in this step, we minimise the cross-entropy loss between the user labels in the training set and values predicted by the user classifier. The user classifier loss function ($L_{u}$) for a single record in a mini-batch is presented in Equation (3). We take the mean loss value of a mini-batch when performing backpropagation. *x* represents an input feature vector of a single record, *y* is a one-hot encoded vector with value of 1 at a corresponding user class label, *E* and $C_{u}$ are the encoder and user classifier networks, respectively, and $N_{u}$ is the number of possible user ID values.

The notation convention remains the same throughout this section, with the index letters representing components related to user classification (*u*) or sensitive-feature classification (*t*).

(3) $$L_{u}(y_{u},C_{u}\left(E\left(x\right)\right)=-\sum_{i=1}^{N_{u}}y_{u_{i}}\;log\left(C_{u}\left(E\left(x_{i}\right)\right)\right)$$

#### 4.4.2. Adversarial Data Flow

In the following step, we train the adversarial flow, i.e., the sensitive-feature classifier, while keeping the encoder fixed. The sensitive-feature classifier infers the hidden feature, providing feedback to the encoder during training and enables us to estimate the encoder’s capability to conceal the information about the hidden feature during testing. Similarly to the user classifier, to train the sensitive-feature classifier, we minimise the value of the cross-entropy loss function (Equation (4)).

(4) $$L_{t}(y_{t},C_{t}\left(E\left(x\right)\right)=-\sum_{i=1}^{N_{t}}y_{t_{i}}\;log\left(C_{t}\left(E\left(x_{i}\right)\right)\right)$$

#### 4.4.3. Encoder Feedback

The last step in the training process relays newly learnt information from the server-based modules to the encoder, teaching it to become a semipermeable mechanism that removes unwanted information while passing relevant data forward. In this training step, we again process the data through the encoder and both server modules and calculate their respective loss functions (Equations (3) and (4)). However, we now negatively weigh the loss function of the sensitive-feature classifier, which consequently guides the encoder to maximise, instead of minimise, the sensitive-feature classifier loss. Complete loss function calculation is presented in Equation (5). We additionally introduce $\lambda_{u}$ and $\lambda_{t}$ that moderate the effect each loss function has on the total feedback to the encoder. We then backpropagate the sum of the gradients from the server modules to the encoder. Note that we do not update the parameters of the task classifier to avoid artificially hurting its performance due to the negatively weighted loss function.

(5) $$L_{e}=\lambda_{u}\times L_{u}-\lambda_{t}\times L_{t}$$

#### 4.4.4. Adversarial Data Flow Replay

In the end, we perform epochs where we only train the sensitive-feature classifier. This training step is uniquely novel to P^2^ESA and, in a sense, emulates a server that conducts the attribute inference attack, i.e., retraining the sensitive-feature classifier after the initial three-step training is over.

After every epoch, we calculate the classification accuracy of the sensitive-feature classifier on the validation part of the dataset. We keep a list of all classification accuracies from this part of the training. We compare the accuracy of the last epoch with the mean value of the list of accuracies with an added ϵ, which we empirically set to 0.01. A lower classification accuracy value of the last epoch indicates that the encoder masks the information well enough to make the attribute inference attack impractical.

If the additional sensitive-feature classifier training leads to high accuracy of sensitive-feature classification, we repeat the three-step training process and the training of the sensitive-feature classifier fine-tuning, as it further boosts the filtering capabilities of the encoder. Otherwise, if the accuracy of the sensitive-feature classifier stays low, the training process is concluded.

## 5. Results

### 5.1. Experimental Setup

We employ two different hardware configurations for the evaluation of P^2^ESA. The first configuration, used for inference accuracy investigation, consists of a server equipped with an Nvidia RTX 3090 24 GB GPU, an AMD 7700x CPU, and 32 GB of memory. In our second configuration, which is used to validate a real-world application, we confirm that the inference accuracy achieved by the first configuration remains stable. Additionally, to measure per-epoch running times of P^2^ESA, we split the training over the above server and an edge device—Lattepanda 3 Delta with an Intel Celeron N5105 CPU and 8 GB of memory. Both the edge device and the server are running the Ubuntu 22.04.3 LTS operating system.

We perform all of our testing within a local network. Both the server and the edge device are connected to the same 1 Gbit/s unmanaged network switch with Cat5e UTP cables and communicate via Ethernet. Such a configuration enables us to minimise potential networking issues while evaluating P^2^ESA. However, we do investigate the impact of a varying connection speed on the model training. For this, we utilise a bash-based bandwidth limiting tool Wondershaper (https://github.com/magnific0/wondershaper, accessed on 16 June 2023), which throttles the connection speed (both upload and download) of the edge device.

### 5.2. Datasets

We validate our system on three IoT datasets. We collected the first two—ca-IoT [6] and MEDBA [27]—as a part of our previous studies. The sensitive-feature that P^2^ESA should obfuscate in these datasets is the current task a person is performing. In another dataset we use Wearable Stress and Affect Detection (WESAD [28]), in which P^2^ESA should obfuscate information about a person’s current emotions.

The ca-IoT dataset includes sensor data of fifteen volunteers performing three office tasks encompassing both computer work and navigation around the office space. The sensing modalities include a six-axis inertial measurement unit (IMU) attached to the top of the keyboard used in the experiments, four force sensors positioned under corners of a rectangular plate that in turn is placed under the keyboard and mouse, six passive infrared (PIR) sensor placed around the office space, and a PC resources monitor, gathering data about CPU, memory, and network usage. The volunteers are between 23 and 58 years old; seven are female, nine are students, and six are full-time employed.

Next, the MEDBA dataset is similar in composition to ca-IoT; however, it includes an optimised set of sensing modalities based on the lessons learned with ca-IoT. PIR sensors are replaced with short-millimetre wave radar placed under the computer monitor, the IMU set is expanded to three units attached to the keyboard, and an additional integrated into the mouse. The positioning of four force sensors remains unchanged, while the PC resources monitor is removed. We utilise data from thirty volunteers aged between 24 and 59 years; twelve are female, and everyone in the study is full-time employed.

The last dataset, WESAD, collects sensor data from two body-worn devices while volunteers were put under different emotional states (neutral, stress, amusement, and baseline). Both devices, chest-worn RespiBAN Professional and wrist-worn Empatica E4, are highly multimodal; however, we focus on the data of integrated IMUs from the two devices. The dataset includes sensor records from fifteen volunteers with a mean age of 27.5 with a standard deviation of 2.4 years, with all participants being students.

All datasets rely on the same data-preprocessing pipeline. First, we apply a sliding window of one second with a half-second overlap to the raw sensor data, which enables us to construct a dataset of uniformly spaced and time-aligned values coming from different sensors. Next, we split the data into a training, validation, and testing set. All datasets include at least two repetitions of all tasks (ca-IoT and MEDBA) or emotional states (WESAD). We utilise one repetition for training, and another for validation and testing, where the first half (time-wise) is used for validation and the second one for testing. Each of the three datasets is then scaled using the MinMaxScaler from the scikit-learn 1.2.0 Python 3.8 package (https://scikit-learn.org/stable/modules/generated/sklearn.preprocessing.MinMaxScaler.html, accessed on 3 May 2023). These steps yield three separate sets of 2-dimensional data with the number of samples and number of sensor signals. Our encoder requests a 3-dimensional input, where the second dimension should consist of *L* consecutive recordings in time, due to the LSTM layer included in the encoder. Therefore, as the last preprocessing step, we assemble all three steps to include this additional dimension with L=7.

### 5.3. Hyperparameter Tuning

We perform two-stage hyperparameter tuning of the neural network model and training variables to establish optimal parameters for the designed architecture. The first stage of the tuning process is performed with a random search, where random values from predetermined value intervals of all parameters are selected. The goal of this stage is to explore wider ranges of parameters and develop intuition on what the final values should be set to. Based on this feedback, we perform the second step of the process, where we rely on a grid parameter search, investigating every possible parameter combination from a much narrower parameter set. The scrutinised parameters, their ranges, and the final selection are presented in Table 2. Both stages are performed on the training and validation datasets. The parameter range of the first stage is set based on previous experience with similar systems. The parameters of the second stage and final values are set based on the achieved F1 scores on the validation dataset.

Additionally, we perform an ablation study on alternative encoder architectures, namely, convolutional neural networks (CNNs), gated recurrent units (GRUs), and long short-term memory (LSTM) architectures. All three encoders are constructed to maximise performance while still fitting within the memory of our edge device. We repeat the training process ten times with each of the architectures on the WESAD dataset and report results in Table 3. The LSTM-based encoder achieves both the highest accuracy and F1 score in terms of user classifier performance and is at least on par with other encoders regarding the sensitive-feature classifier. Given that the running times for different architectures are similar as well, with a maximum per-epoch training delta between encoders of 0.027 s, we opt to use the LSTM-based encoder in P^2^ESA.

### 5.4. Performance Evolution During Training

We first analyse how classification accuracy fluctuates for both user and sensitive-feature classifiers through time and compare it to PAN [7], a similar, privacy-preserving, adversarially trained system. This will help us better understand how the training process takes place and provide insight into why we achieve the results presented later in the section. We train both P^2^ESA and PAN until they achieve their best performance on the WESAD dataset. While the number of actual epochs is different (a PAN epoch is slightly longer due to *k* internal iterations), both algorithms exhibit stable performance at similar times (PAN at 4100 s, P^2^ESA at 4800 s). After the initial training is completed, we re-train the sensitive-feature classifier for an additional 5000 epochs. This attack scenario shines new light on the obfuscating capabilities of the trained encoders of P^2^ESA and PAN.

**Figure 3:**
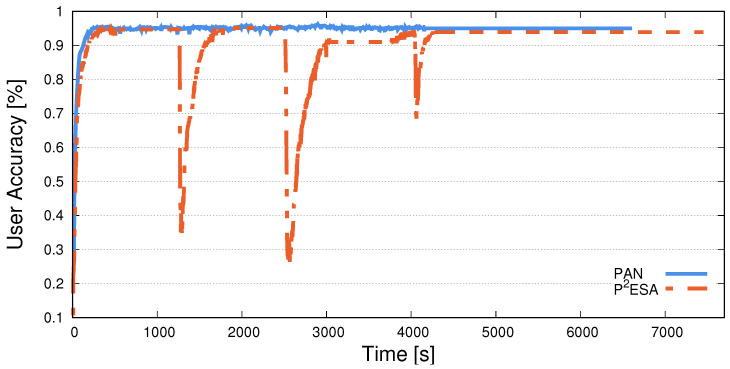
A comparison of the user classification accuracy throughout training for PAN and P^2^ESA on the WESAD dataset. We display the x-axis in time instead of epochs, as an epoch in PAN is much longer due to the nature of the algorithm. The overall run time of both is similar.

Figure 3 displays the change in user classification accuracy through training. Both PAN and P^2^ESA quickly obtain high accuracy, and for PAN, the accuracy remains fairly constant. On the other hand, P^2^ESA experiences dips in accuracy during training. This is because, according to Algorithm 1, P^2^ESA finishes E-epochs of the three training steps (lines 3–16), then re-trains the sensitive-feature classifier for 3 × E epochs (lines 17–25). The above repeats as long as the condition on line 26 is satisfied. With this, P^2^ESA produces an improved sensitive-feature classifier in every iteration of the outer loop (i.e., approx. every 1200–1400 s in Figure 3), while the encoder and the user classifier stay the same. Such a sensitive-feature classifier can provide more valuable feedback to the encoder. This, in turn, allows the encoder to learn to better obfuscate the information about the user’s sensitive feature, lowering the risk of a successful attribute inference attack.

**Figure 4:**
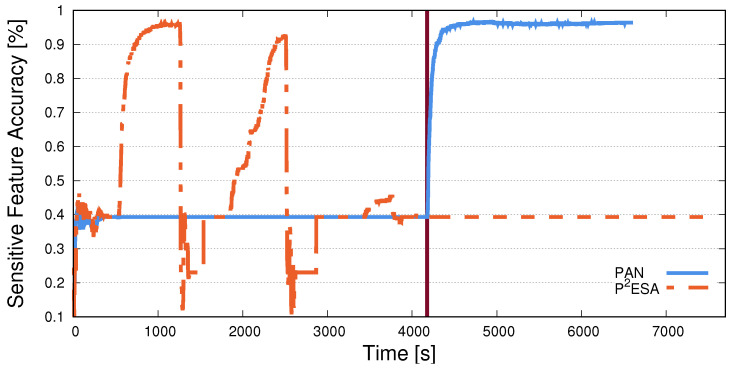
A comparison of the sensitive-feature classification accuracy throughout training for PAN and P^2^ESA. This training is performed on the WESAD dataset—inference of different emotional states. We display the x-axis in time instead of epochs, as an epoch in PAN is much longer, due to the nature of the algorithm. The vertical line represents a moment in time where the adversarial network retrain attack starts. We observe an immediate increase in sensitive-feature classification accuracy for PAN after that point, while values for P^2^ESA stay the same.

In Figure 4, we plot the sensitive-feature inference accuracy with both approaches. We observe a sudden surge in accuracy for PAN. During the initial training, P^2^ESA exhibits a surge in task inference accuracy at times when the sensitive-feature classifier is re-trained. However, once the initial training is completed (around 4800 s), the accuracy remains low even as the attribute inference attack is played out. PAN, on the other hand, gives a false sense of success, as the sensitive-feature inference accuracy remains low during the initial training, yet, completely fails to obfuscate the task once the re-training attack is performed at around 4100 s. This clearly demonstrates how the adversarial data flow replay step introduced in P^2^ESA’s training procedure preserves a user’s privacy.

### 5.5. Trained Models’ Performance

We pit P^2^ESA against a range of alternative approaches for privacy-preserving user authentication and evaluate performance based on classification accuracy and F1 score.

#### 5.5.1. Baseline Approaches

We establish a set of baseline approaches ranging from noise-based augmentation to current state-of-the-art privacy-preserving learning:

- **Separate user and sensitive-feature classifier.** In a form of an ablation study, here we take the encoder and respective classifier parts of P^2^ESA’s architecture and train these parts separately to enable them to perform to the best of their abilities. This establishes an upper limit in terms of performance for both user and sensitive-feature classifiers.
- **Gaussian and Laplace noise** are commonly used data obfuscation techniques [29]. In these baselines, we remove the encoder part of our network and instead add noise on the client side, then train the classifiers according to Algorithm 1. We experiment with different values of standard deviation (Gauss) and exponential decay (Laplace), and set the final testing values at 0.1 and 32. With the presented value set, we invoke setups that focus either on security (high user classification accuracy) or privacy (low sensitive-feature accuracy).
- **Privacy Adversarial Network** is a state-of-the-art, privacy-preserving, adversarial learning technique [7], commonly used as a standard of comparison.

#### 5.5.2. Baseline Comparison

To ensure the robustness of our results, we repeat the training process of each scenario, i.e., a combination of approach and dataset, ten times and report the results in Table 4. The presented results confirm that P^2^ESA is able to simultaneously filter out sensitive feature-related information and retain high user classification accuracy. That in turn enables P^2^ESA to supply encoded data to a third-party authentication system while disabling the ability of the third-party to infer other information from the received data.

Specifically, compared to a privacy-oblivious separately-trained user classifier (i.e., the upper limit of user identification), P^2^ESA performs 5.29% pt and 2.37% pt worse on the ca-IoT and WESAD datasets, respectively, and even outperforms the privacy-oblivious user classifier in MEDBA by 6.70% pt. At the same time, if compared to a separately trained sensitive-feature classifier, P^2^ESA reduces the ability for sensitive-feature inference by 37.26% pt, 59.10% pt, and 28.10% pt for the three datasets. Adding Gaussian or Laplace noise to the data either disables the system’s ability to mask the sensitive feature (standard deviation/exponential decay value of 0.1), or prevents the system from identifying users (standard deviation/exponential decay value of 32), demonstrating that the multi-objective nature of privacy-preserving identification requires a sophisticated encoder provided by P^2^ESA. Compared to PAN, we observe a slight decrease in user classification accuracy achieved by our system: by 0.93% pt and 2.51% pt for the first two datasets, whereas in MEDBA, P^2^ESA performs best overall with a 10.79% pt increase. However, unlike P^2^ESA, PAN struggles to filter out sensitive feature-related information from the data it encodes. In ca-IoT, sensitive-feature classification accuracy reaches 43.22% with PAN, compared to 35.98% with P^2^ESA, in WESAD PAN completely fails to mask sensitive data leading to 95.95% sensitive-feature classification accuracy, compared to 38.36% accuracy achieved with P^2^ESA, and again in MEDBA, where PAN is on par with a separated task classifier, where P^2^ESA sits just above the baseline value (68.05% vs. 39.96%).

### 5.6. Differential Sensitive-Feature Privacy Guarantee

To demonstrate that P^2^ESA achieves robust privacy in terms of a given sensitive feature, we rely on differential privacy (DP) and the Laplace mechanism. We follow the Dwork et al.’s [30] definition of differential privacy, which we apply for P^2^ESA;

(6) $$Pr\left[C_{t}\left(E\left(x\right)\right)\in S\right]\leq e^{\varepsilon }Pr\left[C_{t}\left(E\left(x^{\prime }\right)\right)\in S\right]+\delta$$

where *x* and $x^{\prime }$ are neighbouring datasets, differing in exactly a single row of data. Parameter ε modulates the amount of Laplacian noise introduced in the system, where a smaller ε value guarantees a higher level of privacy, due to more noise being introduced by the system, while δ accounts for a probability of failure. We set δ=0, which results in a (ε,0)-differentially private system in terms of sensitive feature. Finally, selecting a pertinent ε value indicates that any single data sample does not significantly influence the output distribution of $C_{t}\left(E\left(x\right)\right)$ while still maintaining usability, i.e., performance of the user classifier comparable to a system without DP guarantee.

We investigate the effect ε has on the accuracy of our user and sensitive-feature classifiers by analysing it on the MEDBA dataset. After a preliminary analysis, we select two ε values, where one guarantees strong privacy—no leakage of sensitive-feature information, and the other a minimal loss in accuracy of P^2^ESA. After a preliminary test, we select values ε=0.1 and ε=1, respectively.

The results are presented in Table 5. We observe that our strong privacy guarantee ε=0.1 reduces our user classification accuracy by 12.11% pt, reaching performance that is on par with PAN, while simultaneously increasing the obfuscation power of our system as the accuracy of sensitive feature falls by 11.75% pt, compared to our non-DP system. On the other hand, when increasing the privacy budget to facilitate a more accurate but less private system with ε=1, our results are on par with our original, non-DP system.

### 5.7. Running Time Analysis

In a production system, for instance, in an office-like setting, we aim for P^2^ESA to learn iteratively, improving every day by utilising newly gathered data. For this, the system has to be capable of performing a complete (re)training within the time that an office space is not occupied. We assume an eight-hour workday, leaving us with 16 h available for training in a day. Our experimentation shows that P^2^ESA requires at most 2000 epochs for full training (lines 3–16 in Algorithm 1), which leaves us with a maximum 16×60×60 s/2000 = 28.8 s to complete a single epoch training.

In the base configuration, our system achieves an average per-epoch run time of 26.49 s, which is close to our threshold value. We incorporate the Features Replay optimisation technique [31] into our training algorithm to improve the run time. Features Replay assumes that the variance of gradient values between subsequent mini-batches of data is low enough for the algorithm to update the model parameters on stale gradients. This optimisation enables us to, in parallel, perform backpropagation on both edge and server devices. We observe a 9.56 s (26.49 s stock vs. 16.93 s Features Replay) drop in average epoch time while using Features Replay.

**Figure 5:**
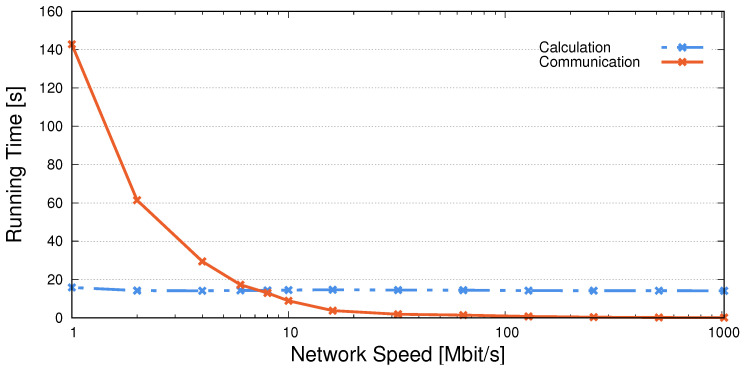
Average training epoch time for networks speeds between 1 Mbit/s and 1 Gbit/s (presented in logarithmic scale). The time it takes to compute a single epoch increases with the decrease in network speed. Calculation running time remains constant. Crosses on both lines represent the actual values used to generate the figure.

Up to this point, we have performed our experiments in almost ideal conditions, as all devices were connected to the same network switch. To investigate how the system would perform in situations where network bandwidth may be a restricting factor, we throttle the network speed of our edge device to different values and train P^2^ESA. We perform 100 epochs and report the average times to complete the calculation part (i.e., calculate both the forward and the backward pass) and the communication part (sending data, labels, and gradients) of a single epoch training. The results are presented in Figure 5. The communication time increases as the network speed decreases, while the calculation time remains constant. To comply with our threshold of 28.8 s, P^2^ESA requires the connection speed to be greater than 4 Mbit/s, which is well below the average global Internet connection speed (https://www.speedtest.net/global-index (accessed on 21 March 2025)) of about 13.5 Mbit/s.

## 6. Discussion

P^2^ESA represents, to the best of our knowledge, the first implementation of a privacy-preserving authentication system that utilises sensor data from IoT environments. The initial results of our experiments are favourable and show that P^2^ESA is successful at masking sensitive information, yet remains capable of identifying users from the encoded data. However, this paper aims to describe the concept and the initial prototype, thus, only a limited amount of effort was spent on performance fine-tuning. Hence, the user identification accuracy is not high enough to allow for stand-alone authentication with P^2^ESA. Instead, we believe P^2^ESA could supplement the existing one-off authentication solutions, such as passwords or fingerprints, by periodically conducting non-intrusive identification and raising an alarm if such continuous authentication fails to confirm the identity of the password-authenticated user. Moreover, the “resolution” of identifiability in a given IoT environment is another aspect that prevents standalone authentication based on behaviour biometrics. For instance, in ca-IoT, we can support only up to 6 users, if identification accuracy above 80% is required, while in the WESAD environment, P^2^ESA can discern among 15 users with over 90% accuracy. Intuitively, there is a limit to uniqueness in human behaviour, and no data processing can overcome it.

Where P^2^ESA shines, however, is in the ability to hide a selected feature. In all three datasets we tested, P^2^ESA was significantly better at obfuscating sensitive data than the state-of-the-art solution PAN and was capable of withstanding the attribute inference attack. While in this paper we considered a single static “hidden” feature whose privacy P^2^ESA was shown to maintain, in certain situations this feature may change over time, say in case the purpose of the smart space changes. In such a case, P^2^ESA can naturally adapt to the new “hidden” feature. As our results from Section 5 show, the full P^2^ESA training pipeline can be completed in less than a day. Merely using a different classifier at the server end (e.g., emotion classifier in place of a task classifier) and retraining the pipeline, makes P^2^ESA prevent a different feature from being leaked.

Feature obfuscation in P^2^ESA is related to secure multi-party computation, as the goal is for the edge device to keep its data private. Nevertheless, unlike the traditional multi-party computation setup, here the roles of the parties are rather asymmetric. The role of the server is to perform the computation (i.e., infer a user’s identity), while the role of the edge device is merely to provide the data. When it comes to trust, it is the server that fully trusts the edge device, while the edge device does not trust the server with the raw data. Consequently, techniques such as homomorphic encryption, garbled circuit, and others are neither needed nor desired, as they would incur unnecessary communication and computation overhead.

Behavioural biometric data can present ethical, legal, and societal challenges while being utilised for authentication purposes, and beyond. Common issues such as bias, fairness, and explainability must be taken into consideration while developing production systems. Tightly related is the legal aspect of such systems, where the developers must follow data protection regulations such as the GDPR [32] in the European Union, and consider potential liability for misuse of such systems by malicious parties, and unfair treatment of legitimate users. While P^2^ESA was designed with the upmost care to ensure that utility and privacy are maximised, the above concerns need to be addressed on a case-by-case basis for each specific setting where P^2^ESA will be used.

## 7. Limitations

Our thorough analysis over three different datasets demonstrates the high potential utility of P^2^ESA in real-world environments. Nevertheless, certain limitations should be taken into account when considering P^2^ESA’s generalisability outside the given datasets.

In P^2^ESA, Algorithm 1 ensures that the encoder is trained to remove sensitive information from the data that flows to the server. However, this process is empirical, and although our experiments with three different datasets indicate its robustness, no formal guarantees about the lack of sensitive information in the encoded data can be given. This potentially makes P^2^ESA vulnerable to attacks that harness machine learning models that are more sophisticated than the sensitive-feature classifier used during the distributed training (Algorithm 1). A related issue is a deliberately under-performing sensitive-feature classifier, or the server deliberately fabricating gradient information during the distributed training, thus negatively affecting the encoder training. Our threat model presented in Section 3 requires that the server complies with the P^2^ESA training protocol and, thus, does not try to deceive by forging the gradient data. Nevertheless, this issue could be countered by firmer user-side control of the training process, for example, by attesting the server-side code and running it within the server’s trusted execution enclave [33]. Similarly, our threat model does not address potential security vulnerabilities within the local environment of the participating IoT devices.

Another limitation comes from the number of users and potential biases of the used datasets that may have affected our results. The datasets we use were collected off participants that do not represent general population, but are likely coming from specific demographics, such as students and academic staff. We further recognise that selected data may be subject to selection, confirmation, and sampling bias. Additionally, there may be confounding variables that we have not accounted for, such as age, disability status, and technology proficiency. While all limitations listed, alongside the empirical nature of our testing methodologies, have an impact on the generalisability of P^2^ESA, it should also be noted that when it comes to a classifier’s ability to identify users, having a more biased population sample (as is the case in this paper) makes the task more difficult; thus, we expect that P^2^ESA will perform no worse on more diverse datasets.

## 8. Conclusions and Future Developments

In this paper, we design and implement P^2^ESA, a system that enables privacy-preserving authentication from data sensed in IoT environments. We propose a novel training algorithm that overcomes the limitations of the current state-of-the-art approaches that are susceptible to the attribute replay attack. We then implement P^2^ESA on an edge device and a server, and optimise it, so that the distributed training can be completed sufficiently fast to allow practical use. Finally, we experiment with three different IoT-sensed datasets and demonstrate that P^2^ESA successfully masks sensitive information, while allowing user authentication on the server side. Full implementation of P^2^ESA is also publicly available at https://gitlab.fri.uni-lj.si/Anc0/pesa, accessed on 4 June 2025.

In the future, we intend to extend our experimental work to include a more advanced threat model that goes beyond inquisitive eavesdropping and includes adversarial environments capable of disrupting training procedures and employing more sophisticated attack methods. Furthermore, we will improve on the underlying performance of user authentication by developing an adaptive authentication system capable of recognising and acting upon different aspects of a user’s behaviour, such as their current mental and physical state, demographics, as well as other external factors, such as time and environmental variables.

## Figures and Tables

**Table 1:** Related privacy obfuscation approaches.

Approach	Data Modalities	Architecture
PAN [7]	Images, Audio Recordings, Inertial Sensors	Convolutional Neural Networks
DeepObfuscator [19]	Images, Text	Convolutional Neural Networks
Olympus [20]	Images, Inertial Sensors	Convolutional Neural Networks
Blinder [21]	Variational	Convolutional Neural Networks

**Table 2:** Hyperparameters and their defined ranges for each step of the hyperparameter-tuning process. The final value of some parameters was already set after Stage 1; those that only have a single value are noted already in Stage 2.

Parameter	Stage 1	Stage 2	Final
epochs	100–10,000	100–1000	500
batch size	$2^{2}$–$2^{12}$	$2^{10}$–$2^{12}$	$2^{11}$
learning rate	10 × 10^−2^–10 × 10^−5^	10 × 10^−4^	10 × 10^−4^
dropout	0.25–0.75	0.25–0.5	0.3
LSTM encoder size	$2^{5}$–$2^{10}$	$2^{6}$–$2^{8}$	$2^{7}$
dense encoder size	$2^{5}$–$2^{10}$	$2^{6}$–$2^{8}$	$2^{7}$
dense classifier size 1	$2^{5}$–$2^{10}$	$2^{5}$–$2^{7}$	$2^{6}$
dense classifier size 2	$2^{4}$–$2^{9}$	$2^{4}$–$2^{6}$	$2^{5}$
epoch ratio	1:1–1:5	1:3	1:3
λ_u	0.1–1	1	1
λ_t	0.1–1	0.1–0.5	0.1

**Table 3:** Ablation study of different encoder architectures assessing their ability to properly identify users and successfully hide sensitive features. Three out of four best results of each column (marked with bold) correspond to the LSTM architecture. Note that the training times are similar across the architectures.

Architecture	User Acc	Feat Acc	User F1	Feat F1
CNN	89.27 ± 3.89%	38.59 ± 7.24%	89.59 ± 4.37%	24.66 ± 7.23%
GRU	92.04 ± 2.81%	**38.16** ± 8.48%	92.19 ± 3.34%	22.07 ± 7.72%
LSTM	**93.31** ± 2.8%	38.36 ± 8.76%	**93.27** ± 2.53%	**21.27** ± 8.04%

**Table 4:** Classification accuracy (acc) and F1 scores for user and sensitive-feature classifiers of P^2^ESA, PAN, a separate user classifier (user) and separate sensitive-feature classifier (feat), and Gaussian (G) and Laplace (L) noise with different scale values after the training and the attribute inference attack are conducted. We observe that P^2^ESA is able to obfuscate sensitive feature-related information, while maintaining a high user classification accuracy and F1 score. Baseline is the majority classifier, and ± values are the standard deviation of the achieved results over ten runs.

Approach	User Acc	Feat Acc	User F1	Feat F1
ca-IoT dataset				
baseline	6.67%	33.33%	6.67%	33.33%
user	**81.14** ± 3.42%	NA	**79.98** ± 4.79%	NA
feat	NA	73.24 ± 6.81%	NA	73.19 ± 6.02%
G 0.1	44.12 ± 3.75%	48.96 ± 2.3%	45.3 ± 2.15%	48.74 ± 3.79%
L 0.1	38.49 ± 4.24%	47.52 ± 4.41%	37.48 ± 4.95%	47.13 ± 3.21%
G 32	7.1 ± 1.36%	32.73 ± 5.5%	1.28 ± 1.54%	19.2 ± 3.94%
L 32	7.19 ± 0.93%	**32.32** ± 5.12%	1.36 ± 1.87%	**16.1** ± 2.49%
PAN	76.78 ± 8.84%	43.22 ± 2.75%	76.05 ± 6.65%	43.30 ± 4.34%
P^2^ESA	75.85 ± 9.85%	35.98 ± 2.94%	73.87 ± 6.35%	37.41 ± 4.51%
WESAD dataset				
baseline	6.67%	25%	6.67%	25%
user	95.68 ± 3.77%	NA	95.66 ± 3.3%	NA
feat	NA	97.46 ± 1.21%	NA	97.46 ± 0.86%
G 0.1	71.66 ± 3.6%	83.64 ± 2.93%	68.31 ± 4.45%	82.2 ± 3.03%
L 0.1	67.19 ± 3.11%	83.45 ± 2.82%	61.99 ± 4.41%	82.36 ± 3.84%
G 32	6.07 ± 1.74%	38.82 ± 2.61%	3.93 ± 1.14%	21.72 ± 3.11%
L 32	6.93 ± 2.21%	38.82 ± 3.74%	3.95 ± 2.55%	21.71 ± 4.16%
PAN	**95.82** ± 2.24%	95.95 ± 1.62%	**95.81** ± 2.45%	95.94 ± 1.63%
P^2^ESA	93.31 ± 2.8%	**38.36** ± 8.76%	93.27 ± 2.53%	**21.27** ± 8.04%
MEDBA dataset				
baseline	3.33%	33.33%	3.33%	33.33%
user	74.59 ± 3.26%	NA	69.28 ± 3.12%	NA
feat	NA	68.06 ± 6.01%	NA	67.94 ± 6.45%
G 0.1	56.89 ± 2.16%	55.22 ± 2.31%	55.32 ± 2.27%	54.49 ± 2.36%
L 0.1	57.34 ± 3%	60.43 ± 2.21%	58.55 ± 3.31%	58.48 ± 2.49%
G 32	6.6 ± 1.28%	**33.49** ± 2.67%	3.22 ± 1.87%	34.96 ± 3.17%
L 32	5.7 ± 0.77%	33.63 ± 2.48%	3.85 ± 0.96%	**34.72** ± 2.75%
PAN	70.59 ± 6.01%	68.05 ± 2.68%	67.52 ± 6.48%	67.81 ± 2.47%
P^2^ESA	**81.38** ± 7.14%	39.96 ± 8.62%	**78.84** ± 7.93%	38.62 ± 9.79%

**Table 5:** Classification accuracy (acc) and F1 scores for user and sensitive-feature classifiers of P^2^ESA on the MEDBA dataset. We apply different ε values to form a strong privacy guarantee and establish the tradeoff in loss of performance, compared to privacy.

Approach	User Acc	Feat Acc	User F1	Feat F1
no DP	**81.38** ± 7.14%	39.96 ± 8.62%	**78.84** ± 7.93%	38.62 ± 9.79%
ε 0.1	69.27 ± 6.77%	**28.21** ± 6.8%	67.72 ± 7.19%	**29.11** ± 6.91%
ε 1	81.12 ± 6.81%	40.28 ± 7.14%	78.61 ± 6.42%	39.44 ± 7.48%

## Data Availability

The original data presented in the study are openly available at https://ubi29.informatik.uni-siegen.de/usi/data_wesad.html (WESAD), https://gitlab.fri.uni-lj.si/lrk/ca-iot (ca-IoT), and https://data.jrc.ec.europa.eu/dataset/7be86ffd-1bac-4d3e-82fc-02b3ea40ab49 (MEDBA) all accessed on 4 August 2025.
